# Supplementary material for: An optimized initialization for LDPC decoding over GF(q) in impulsive noise environments
Source: PLoS One. 2021 May 6;16(5):e0250930. doi: 10.1371/journal.pone.0250930 (PMC8101722; doi:10.1371/journal.pone.0250930)
Supplement: Attachment — Submitted filename: Response_to_Reviewers.doc [file pone.0250930.s003.doc]

**Original Manuscript Number:** PONE-D-20-15556

**Original Article Title:** “An optimized initialization for LDPC decoding over GF(*q*) in impulsive noise environments”

**To:** PLOS ONE Editor

**Re:** Response to reviewers

Dear Editor,

Thank you for allowing a resubmission of our manuscript, with an opportunity to address the reviewers’ comments. We would like to thank the Editor and the reviewers for their valuable comments and feedback that has helped us make improvements to this paper. We have carefully revised the article and now updated the paper addressing all of the reviewer comments. Below we have carefully answered all of the reviewers’ questions and comments.

We are uploading (a) our point-by-point response to the comments (below) (response to reviewers), (b) an updated manuscript with highlighting indicating changes, and (c) a clean updated manuscript without highlights (PDF main document).

The formatting is as follows:

*In this part of the paper:*

A change, that I highlight, is made while this is added.

Best regards,

Hongbo Zhao, et al.

bhzhb@buaa.edu.cn

**Additional Editor Comments (if provided):** The paper seems to be revised well. All the comments are addressed. There is one minor revisions to be noticed. LDPC decoding is a very old technique, so please add some new background and references for that, like 5G or 6G application related references. For example, [1] "Massively Distributed Antenna Systems with Nonideal Optical Fiber Fronthauls: A Promising Technology for 6G Wireless Communication Systems," IEEE Vehicular Technology Magazine, Dec. 2020, [2] "What should 6G be?" Nature Electronics, Jan. 2020; and so.

**Author response:** Thanks for this comment very much. The authors agree that there should be more background and references related to 5G or 6G application in our manuscript. As LDPC decoding is an old technique, we hope to highlight the connection between this paper and the latest communication system.

**Author action:** We have made the manuscript more clearly. We updated by including some new background in Section 1 “Introduction” and adding some related references to emphasize the 5G or 6G application as follows:

With the explosive development of communication technology, the new mobile communication systems, such as beyond fifth generation (B5G) and sixth generation (6G) systems, will suffer from severe challenges imposed by the requirement for heavy connection density and high efficiency [1]. Especially for 6G, satellite communications play an important role in providing high quality communication services to achieve the worldwide connectivity [2]. As one of the critical components, …

[1].	Yu L, Wu J, Zhou A, et al. Massively Distributed Antenna Systems With Nonideal Optical Fiber Fronthauls: A Promising Technology for 6G Wireless Communication Systems. IEEE Vehicular Technology Magazine. 2020; 15(4): 43-51.

[2].	Dang S, Amin O, Shihada B, et al. What should 6G be?. Nature Electronics. 2020; 3(1): 20-29.

Further, in Section 6 “Conclusion”, we have included the consideration of the next communication systems as follows:

The optimized initialization proposed in this paper can be also extended to decoding process of other 5G or 6G applications. In the future work, we will focus on studying LDPC decoding over GF(q) in a more complex noise environment for B5G and 6G systems.
